# Supplementary material for: Provide energy-aware routing protocol in wireless sensor networks using bacterial foraging optimization algorithm and mobile sink
Source: PLoS One. 2022 Mar 23;17(3):e0265113. doi: 10.1371/journal.pone.0265113 (PMC8942282; doi:10.1371/journal.pone.0265113)
Supplement: Attachment — Submitted filename: Comments and Suggestions for Authors _PONE.pdf [file pone.0265113.s002.pdf]

## Comments and Suggestions for Authors

The article describes a new algorithm is proposed that clusters sensor nodes in wireless sensor networks using the bacterial foraging optimization algorithm to improve the energy consumption problem.

Major comments:

It seems there is much room for improvement before the publication. I list my major comments on the manuscript below.

1- The innovation and the motivation behind this work are not clearly highlighted. Please work on this and prove to us why this work is valuable. And the novelty of the proposed model is questionable.
2- The contributions and novelty of this paper are not listed in the introduction section.
3- On page 8, the claim (Wireless sensor networks (WSNs) include small sensor nodes with battery and processing power and limited memory units. There are two important features of WSNs, one is infrastructure-free and the other is self-organizing.) need to be supported by references.
4- The authors used a number of abbreviations without clarification the original words of these abbreviations, for example, but not limited to "DCRRP", "OPNET", "SFLA", and "FIS"
5- On page 9, the claim (The advantage of this method is the use of an optimized aggressive weed algorithm that can adapt and be random and can quickly find the global optimal, as well as the use of a fuzzy inference model that has high accuracy. The disadvantage of this method is the high processing cost due to the use of fuzzy logic with an aggressive weed algorithm.) need to be supported by references.
6- The reference [8] is missed in the text.
7- OPNET 11.5 Simulator and BIFOA need references.
8- In general, The values of parameters (for example, s, p, $W_{attractant}$, $d_{attractant}$ , $w_{repllant}$, $d_{repllant}$, the number of sensor nodes is 60, and the values in table 1 ) were selected arbitrarily. The authors provide no guidance on how and why to select these values, no references supported that.
9- Correct the writing ith, jth, and kth. Also, correct hrepllant → $h_{repllant}$ and $W_{replant}$ → $W_{repllant}$.
10- Equations1 and 2 need references.

11- In this sentence (In this regard, $W_{repllant}$, $W_{attractant}$, $W_{attractant}$, $d_{attractant}$ are different coefficients that must be selected correctly.) $W_{attractant}$ is repeated, maybe it must change to $h_{repllant}$ .

12- On page 14, where is Equation 3-2?

13- On page 14, the claim (However, because the $W_{attractant}$ value is always considered less than the $W_{repllant}$.) why? And needs to be supported by references.

14- In Figure 1, the direction of the arrow in the loop is needed.

15- Adjust Equations 3 and 4. Also, adjust the format of Table 1.

16- On page 16, step 2, ie → i.e.

17- On page 16, step 3, selects fifty percent, why? It needs a reference.

18- On page 17, step 4, ped → $P_{ed}$

19- In step 6, if there is a malicious node, and participated in the process, what the effect on the proposed method, and how it treats this issue?

20- On page 17, (If the residual energy of the sprigs is more than 70% of their total initial energy,). Why you selected this value? This claim needs to support by references.

21- On page 21, 3-4 Simulation results → 4-3 Simulation results.

22- On page 21, simulation results subsection, on line 8, there is a comma (,) it must remove it.

23- What are the mobility and energy consumption models used in this paper?

24- Platform configuration and operating system are used to test run are also not stated very well.

25- In Figure 5, the values of the two methods are approximately similar, is that mean the proposed method not efficient when the value of pause time is high?

26- In Figures 5, and 6, the horizontal axis shows the simulation time or the pause time?

27- In Figure 6, the values of end-to-end delay are approximately similar for all pause time values, is that mean end-to-end delay not affected by pause time? If yes, why you study it?

28- The results in Figures 6 and 7 are questionable because when the value of pause time is small that means the mobility of the nodes in the network is high, so the probability to break the links between nodes will increase. As a result, the throughput will decrease and the delay will increase.
