# Supplementary material for: Provide energy-aware routing protocol in wireless sensor networks using bacterial foraging optimization algorithm and mobile sink
Source: PLoS One. 2022 Mar 23;17(3):e0265113. doi: 10.1371/journal.pone.0265113 (PMC8942282; doi:10.1371/journal.pone.0265113)
Supplement: Attachment — Submitted filename: Response to Reviewers_tabataabei.doc [file pone.0265113.s003.doc]

**September 02, 2021**

**Dear Dr. Ping He,**

**Re your email, I am writing to submit the revised version of the manuscript entitled “Provide energy-aware routing protocol in wireless sensor networks using bacterial foraging optimization algorithm and mobile sink	” (ID: PONE-D-21-15940	).**

**I would like to take this opportunity to express my appreciation for the invaluable comments and suggestions which you and the reviewer kindly provided. I tried my best to revise the manuscript in accordance with the comments. Detailed corrections are listed below point by point. Also, I have highlighted the changes I made in the manuscript. I hope the revised form of the manuscript meets the expectation of the reviewers and is suitable for publication in “PLOS ONE.”**

**Thank you again for your consideration.**

**I am looking forward to reading from you.**

**Sincerely yours,**

**Dr. Sh.Tabatabaei**

**………………………………………………………………………………………………………**

**Responses to Comments**

**Reviewer**

| **#** | **Comment** | **Response** |
| --- | --- | --- |
| **Reviewer #1** | | |
| **1** | **The innovation and the motivation behind this work are not clearly highlighted. Please work on this and prove to us why this work is valuable. And the novelty of the proposed model is questionable.** | **Correction was made and added innovation and the motivation this work in introduction.** |
| **2** | **The contributions and novelty of this paper are not listed in the introduction section** | **Correction was made.** |
| **3** | **On page 8, the claim (Wireless sensor networks (WSNs) include small sensor nodes with battery and processing power and limited memory units. There are two important features of WSNs, one is infrastructure-free and the other is self-organizing.) need to be supported by references.** | **Correction was made and the reference is added in the text.** |
| **4** | **The authors used a number of abbreviations without clarification the original words of these abbreviations, for example, but not limited to “DCRRP”, “OPNET”, “SFLA”, and “FIS”** | **The original words of abbreviations“DCRRP”, “OPNET”, “SFLA”, and “FIS” are added.** |
| **5** | **On page 9, the claim (The advantage of this method is the use of an optimized aggressive weed algorithm that can adapt and be random and can quickly find the global optimal, as well as the use of a fuzzy inference model that has high accuracy. The disadvantage of this**  **method is the high processing cost due to the use of fuzzy logic with an aggressive weed algorithm.) need to be supported by references.** | **Correction was made and the reference is added in the text.** |
| **6** | **The reference [8] is missed in the text.** | **The reference [8] is added in the text.** |
| **7** | **OPNET 11.5 Simulator and BIFOA need references.** | **Correction was made and the reference [24] is added for OPNET 11.5 also the reference [22] is added for BIFOA.** |
| **8** | **In general, The values of parameters (for example, s, p, Wattractant, dattractant , wrepllant, drepllant, the number of sensor nodes is 60, and the values in table 1 ) were selected arbitrarily. The authors provide no guidance on how and why to select these values, no references supported that.** | **The simulation result of the proposed protocol is compared with the AFSRP (Artificial Fish Swarm Routing Protocol) then all parameter is similar with the reference [21]. Also, the reference [21] is added for the parameter in table 1.** |
| **9** | **Correct the writing ith, jth, and kth. Also, correct hrepllant -> h_repllant_ and Wreplant -> W_repllant_.** | **Correction was made.** |
| **10** | **Equations1 and 2 need references.** | **Correction was made and Equations 1 and 2 are referenced with [22].** |
| **11** | **In this sentence (In this regard, Wrepllant, Wattractant, Wattractant, dattractant are different coefficients that must be selected correctly.) Wattractant is repeated, maybe it must change to hrepllant .** | **Correction was made and Wattractant changed to hrepllant .** |
| **12** | **On page 14, where is Equation 3-2?** | **Correction was made and Equation 3-2 changed to Equation 2.** |
| **13** | **On page 14, the claim (However, because the Wattractant value is always considered less than the Wrepllant.) why? And needs to be supported by references.** | **Correction was made and the reference is added.** |
| **14** | **In Figure 1, the direction of the arrow in the loop is needed.** | **The direction of the arrow in the loop is added in Figure 1.** |
| **15** | **Adjust Equations 3 and 4. Also, adjust the format of Table 1.** | **Correction was made.** |
| **16** | **On page 16, step 2, ie -> i.e.** | **Correction was made.** |
| **17** | **On page 16, step 3, selects fifty percent, why? It needs a reference** | **Because in the bacterial foraging optimization algorithm selects fifty percent of the bacteria, then we selects fifty percent in step 3, reference is added.** |
| **18** | **On page 17, step 4, ped -> Ped.** | **Correction was made.** |
| **19** | **In step 6, if there is a malicious node, and participated in the process, what the effect on the proposed method, and how it treats this issue?** | **With consideration, that the sink has a table content of IDs of all sensor nodes and the sink sends a notification message of being cluster head to cluster head sensors, then if malicious node want to participate in the process, sink detected it, and sends IDs of malicious node for all selected cluster head to not accept join request from this malicious node.** |
| **20** | **On page 17, (If the residual energy of the sprigs is more than 70% of their total initial energy,). Why you selected this value? This claim needs to support by references.** | **Correction was made and the reference [9] is added. This sentence" If the residual energy of the sprigs is more than 70% of their total initial energy "is in reference [9].** |
| **21** | **On page 21, 3-4 Simulation results -> 4-3 Simulation results.** | **Correction was made.** |
| **22** | **On page 21, simulation results subsection, on line 8, there is a comma (,) it must remove it.** | **Correction was made and comma (,) removed.** |
| **23** | **What are the mobility and energy consumption models used in this paper?** | **The mobility model is random, it is added to table 1 and energy consumption equation number 6 is added in the text.** |
| **24** | **Platform configuration and operating system are used to test run are also not stated very well.** | **Platform configuration is show in fig 4 and we use Contiki operating system that it uses standard C and follows an event-driven programming model that added in the text.** |
| **25** | **In Figure 5, the values of the two methods are approximately similar, is that mean the proposed method not efficient when the value of pause time is high?** | **The proposed method is efficient for all constant time or all simulation, also it is efficient when the value of time is high in figure 5 (energy consumption) shows only one pause time may the proposed method and the AFSRP method is maybe similar.** |
| **26** | **In Figures 5, and 6, the horizontal axis shows the simulation time or the pause time?** | **In Figures 5, and 6, the horizontal axis shows the pause time.** |
| **27** | **In Figure 6, the values of end-to-end delay are approximately similar for all pause time values, is that mean end-to-end delay not affected by pause time? If yes, why you study it?** | **Attention in Figure 6, for the proposed method the values of end-to-end delay for all pause time and all simulation time value is efficient and better than the AFSRP method.** |
| **28** | **The results in Figures 6 and 7 are questionable because when the value of pause time is small that means the mobility of the nodes in the network is high, so the probability to break the links between nodes will increase. As a result, the throughput will decrease and the delay will increase** | **in proposed method all nodes is fixed and no mobility for the nodes only sink is mobiled in the network, other wise the sink will make a decision based on the residual energy of the sprigs in its table, If the residual energy of the sprigs is less than 70%, the sink moves to a denser area then the cluthe probability to break the links between nodes will decrease. As a result, the throughput will increase. also with the moves of sink toward denser area the cluster heads can send data with the nearest way to sink then decrease delay.** |

**………………………………………………………………………………………………………**
