# Supplementary material for: Provide energy-aware routing protocol in wireless sensor networks using bacterial foraging optimization algorithm and mobile sink
Source: PLoS One. 2022 Mar 23;17(3):e0265113. doi: 10.1371/journal.pone.0265113 (PMC8942282; doi:10.1371/journal.pone.0265113)
Supplement: Attachment — Submitted filename: Response to Reviewers_tabataabei2.doc [file pone.0265113.s004.doc]

**December 11, 2021**

**Dear Dr. Ping He,**

**Re your email, I am writing to submit the revised version of the manuscript entitled “Provide energy-aware routing protocol in wireless sensor networks using bacterial foraging optimization algorithm and mobile sink	” (ID: PONE-D-21-15940R1	).**

**I would like to take this opportunity to express my appreciation for the invaluable comments and suggestions which you and the reviewer kindly provided. I tried my best to revise the manuscript in accordance with the comments. Detailed corrections are listed below point by point. Also, I have highlighted the changes I made in the manuscript. I hope the revised form of the manuscript meets the expectation of the reviewers and is suitable for publication in “PLOS ONE.”**

**Thank you again for your consideration.**

**I am looking forward to reading from you.**

**Sincerely yours,**

**Dr. Sh.Tabatabaei**

**………………………………………………………………………………………………………**

**Responses to Comments**

Reviewer

| **#** | **Comment** | **Response** |
| --- | --- | --- |
| **Reviewer #3** | | |
| **1** | **The contributions and novelty of this paper are not listed in the introduction section.** | **A correction was made and added contributions and novelty to this work in sections 1-1 of the introduction.** |
| **2** | **Review the paper to be sure the font is the same for all the paper.** | **The correction was made.** |
| **3** | **Adjust Equations1 and 2, also put their references in a suitable place. Also, for Equation 6.** | **The correction was made and adjusted Equations 1,2 and 6.** |
| **4** | **Adjust Equations 3 and 4.** | **The correction was made and adjusted Equations 3 and 4.** |
| **Reviewer #4** | | |
| **1** | **Check the English grammar mistakes and correct them.** | **The correction was made.** |
| **2** | **The introduction section is a single paragraph, which makes it difficult to read. Try to divide it into several paragraphs.** | **The correction was made and the introduction section was divided into several paragraphs.** |
| **3** | **At the end of the introduction section, the contribution list should be included and stated clearly.** | **The correction was made and the contribution list be included and stated clearly.** |
| **4** | **In the related work section, the recent algorithm needs to be included such as: [I] A. S. Al-Zubaidi, et al. “Re-evaluation of the stable improved LEACH routing protocol for wireless sensor network,” in Proceedings of the International Conference on Information and Communication Technology - ICICT ’19, 2019, pp. 96–101. [II] Salem, Amer O. Abu, and Noor Shudifat. "Enhanced LEACH protocol for increasing a lifetime of WSNs." Personal and Ubiquitous Computing 23.5 (2019): 901-907.** | **The correction was made and this reference is added in [23] and [22].** |
| **5** | **The paragraphs need to be divided into several paragraphs for more readability.** | **The correction was made.** |
| **6** | **A table need to be included at the end of the related work section. The table summarizes the algorithm in the section as well as includes the highlights and limitations of each algorithm.** | **The correction was made and added the table summarized each algorithm.** |
| **7** | **symbol should be included in math mode not in regular text mode.** | **The correction was made.** |
| **8** | **some equation are bold and not aligned correctly . please correct them.** | **The correction was made.** |
| **9** | **each variable in the manuscript need to be defined clearly.** | **Correction was made and each variable in the manuscript need is idefined clearly.** |
| **10** | **Try not to use “_”, the subscript can be used.** | **The correction was made and used the subscript.** |
| **11** | **Figures 1 need to be enlarged. In addition, the text is not clear inside the blue color, try to select clear colors.** | **Correction was made and Figures 1 was enlarged and selected clear colour.** |
| **12** | **Figure 3 should be replaced by algorithm style. In addition, the algorithm is not clear and not given in a professional manner.** | **Correction was made.** |
| **13** | **more discussion about the results should be included.** | **Correction was made and more discussion about the results added.** |
| **14** | **missing state of the art algorithm in the comparison . Try to include some .** | **Correction was made.** |
| **Reviewer #5** | | |
| **1** | **Too lengthy abstract. too many introduction.** | **Correction was made and changed abstaract also the introduction section was divided into several paragraphs.** |
| **2** | **Check the literature After 2019.** | **Correction was made and the reference [22] and [23] is added.** |
| **3** | **Introduction section may split two or three paragraphs.** | **Correction was made and the introduction section was divided into several paragraphs.** |
| **4** | **Introduction must summarize the existing method's limitations. and then your highlighted contributions.** | **the correction was made and added the summary of the existing methods is in table 1.** |
| **5** | **Figure 2 is similar to table?** | **Figure 2 is not table.** |
| **6** | **Equations should be properly edited. (i.e) 3, 4 etc** | **Correction was made.** |
| **7** | **Figure 3 should be typed version. Not In the present format.** | **The correction was made and Figure 3 is changed.** |
| **8** | **Since the energy is major focus of your work**  **- Analyse should include energy vs no of nodes /Analyse should include energy vs no of sensors**  **- Analyse should include energy vs distance**  **- Analyse should include energy vs simulation time** | **All charts of analysing include simulation time.**  **And the major focus of my work is to increase lifetime in WSN also all charts such as an end to end delay, throughput, energy consumption, success data transfer and signal to noise ratio is showed.** |
| **9** | **Illustration of your work or general block diagram of your approach must be given** | **The Fig 1 showed the flowchart of my algorithm** |

**………………………………………………………………………………………………………**
